# Lovastatin for the Treatment of Adult Patients With Dengue: A Randomized, Double-Blind, Placebo-Controlled Trial

**DOI:** 10.1093/cid/civ949

**Published:** 2015-11-12

**Authors:** James Whitehorn, Chau Van Vinh Nguyen, Lam Phung Khanh, Duong Thi Hue Kien, Nguyen Than Ha Quyen, Nguyen Thi Thanh Tran, Nguyen Thuy Hang, Nguyen Thanh Truong, Luong Thi Hue Tai, Nguyen Thi Cam Huong, Vo Thanh Nhon, Ta Van Tram, Jeremy Farrar, Marcel Wolbers, Cameron P. Simmons, Bridget Wills

**Affiliations:** 1 London School of Hygiene and Tropical Medicine, United Kingdom; 2 Oxford University Clinical Research Unit; 3 Hospital for Tropical Diseases, Ho Chi Minh City; 4 Tien Giang Hospital, My Tho, Vietnam; 5 Centre for Tropical Medicine and Global Health, Nuffield Department of Clinical Medicine, Oxford University, United Kingdom; 6 Department of Microbiology and Immunology, Peter Doherty Institute, University of Melbourne, Victoria, Australia

**Keywords:** dengue, lovastatin, randomized clinical trial

## Abstract

***Background.*** Dengue endangers billions of people in the tropical world, yet no therapeutic is currently available. In part, the severe manifestations of dengue reflect inflammatory processes affecting the vascular endothelium. In addition to lipid lowering, statins have pleiotropic effects that improve endothelial function, and epidemiological studies suggest that outcomes from a range of acute inflammatory syndromes are improved in patients already on statin therapy.

***Methods.*** Following satisfactory review of a short pilot phase (40 mg lovastatin vs placebo in 30 cases), we performed a randomized, double-blind, placebo-controlled trial of 5 days of 80 mg lovastatin vs placebo in 300 Vietnamese adults with a positive dengue NS1 rapid test presenting within 72 hours of fever onset. The primary outcome was safety. Secondary outcomes included comparisons of disease progression rates, fever clearance times, and measures of plasma viremia and quality of life between the treatment arms.

***Results.*** Adverse events occurred with similar frequency in both groups (97/151 [64%] placebo vs 82/149 [55%] lovastatin; *P* = .13), and were in keeping with the characteristic clinical and laboratory features of acute dengue. We also observed no difference in serious adverse events or any of the secondary outcome measures.

***Conclusions.*** We found lovastatin to be safe and well tolerated in adults with dengue. However, although the study was not powered to address efficacy, we found no evidence of a beneficial effect on any of the clinical manifestations or on dengue viremia. Continuing established statin therapy in patients who develop dengue is safe.

***Chinese Clinical Trials Registration.*** ISRCTN03147572.

## Abstract (precis)

Dengue is a viral disease for which there is currently no therapeutic agent. We investigated the potential of lovastatin in the treatment of dengue. Lovastatin was safe and well tolerated, but did not demonstrate a therapeutic benefit.

Dengue exerts an enormous toll on the tropical world, with approximately 390 million infections annually [1]. While most patients recover after a short but often debilitating illness, a proportion progress to severe disease, characterized by endothelial dysfunction and plasma leakage that may result in hypovolemic shock [2]. Other potentially severe complications include bleeding and liver/organ involvement [2]. Myalgia and arthralgia are prominent symptoms associated with dengue, and rhabdomyolysis is also occasionally reported [3]. No vaccine or effective therapeutic is available as yet, and health systems in endemic areas are frequently overwhelmed with patients during the dengue season [2]. There is a need for a therapeutic that can shorten the duration and severity of symptoms.

The 3-hydroxy-3-methylglutaryl-coenzyme A (HMG-CoA) reductase inhibitors, also known as statins, have beneficial effects beyond their lipid-lowering properties, including effects on endothelial function [4, 5]. These effects are mediated through effects on the mevalonate pathway, resulting in reduced expression of proinflammatory cytokines, thereby controlling leukocyte migration to areas of endothelial inflammation [6]. Statins may improve endothelial function by modulating the production of reactive oxygen species [7]. Endothelial dysfunction is recognized as an important factor in atherosclerosis pathogenesis [8], and these effects, together with the lipid-lowering properties, likely explain the benefits of statin use in cardiovascular disease. There is overlap between the inflammatory processes seen in atherosclerosis and sepsis, and observational studies have suggested that statin therapy may be associated with improved outcomes in inflammatory conditions [9–11]. Statins are being investigated as adjunctive therapy for a variety of conditions such as sepsis and acute respiratory distress syndrome in which endothelial dysfunction is thought to play a role. To date their adjunctive role has not been substantiated [12, 13].

Although the mechanisms underlying the endothelial dysfunction seen in severe dengue remain incompletely understood, we hypothesized that the endothelial stabilizing effects of statins could favorably modulate dengue vasculopathy. In addition, in vitro work has shown that lovastatin reduces dengue virion assembly, raising the possibility that statins may have antiviral properties [14–16]. Although statins have a good safety profile, given the recognized association between statin use and hepatic and muscle dysfunction [17, 18], both well-recognized features of dengue, our primary objective was to assess the safety of lovastatin in dengue patients. In addition, we investigated the effects of lovastatin on a variety of clinical and virological parameters.

## METHODS

### Study Design and Participants

We performed a randomized, placebo-controlled, double-blind, dose-escalating trial of lovastatin for adult dengue patients at 2 centers in Vietnam (Hospital for Tropical Diseases, Ho Chi Minh City; Tien Giang Hospital, My Tho City) [19]. In phase 1, we assessed 40 mg lovastatin vs placebo in 30 patients, followed, after satisfactory safety review, by 80 mg lovastatin vs placebo in 300 patients in phase 2.

Ethical approval was obtained from the institutional review boards (IRBs) of the Hospital for Tropical Diseases and the Vietnam Ministry of Health, the London School of Hygiene and Tropical Medicine, and the Oxford University Tropical Research Ethics Committee. The trial was registered with the ISRCTN registry (ISRCTN03147572).

The trial protocol has been published elsewhere [19]. In brief, patients aged ≥18 years, presenting within 72 hours of fever onset with an illness consistent with dengue, in whom a rapid test for dengue nonstructural protein 1 (NS1) was positive (NS1 Ag-STRIP, Bio-Rad) were eligible for inclusion providing they gave written informed consent. Exclusions included an alanine aminotransferase (ALT) level >150 U/L, a creatine kinase (CK) level >1000 U/L, a platelet count <50 cells × 10^9^/L, pregnancy or lactation, or a history of cirrhosis or myopathy. Patients were excluded if they were currently taking statins or any medication contraindicated with statins.

### Randomization and Masking

We randomly assigned enrolled patients in a 1:1 ratio to receive lovastatin or placebo once daily for 5 days. Randomization was stratified by ward of recruitment, using block randomization (block sizes of 4 or 6). The Vietnamese company DOMESCO provided lovastatin and visually matched placebo without charge. Only the clinical trials pharmacist had access to the randomization list; she prepackaged bottles containing 6 doses of either lovastatin or visually matched placebo. All other study staff were blinded to treatment allocation until after completion of the study and database locking. Each enrolled patient was assigned the next available study code that corresponded to a prepackaged bottle of study drug. Clinical data were captured on structured case report forms that were entered into a secure Web-based database.

### Study Procedures

Participants were identified on the dengue wards and among patients attending outpatients who agreed to be hospitalized. Screening was streamlined to ensure completion within 4 hours. Participants received the first dose of study drug as soon as possible after randomization, with subsequent doses given once daily in the morning. Standardized clinical information was recorded daily throughout the disease course. Study physicians were responsible for all clinical management decisions with the following caveats: If the ALT level exceeded 250 U/L, the CK level exceeded 1000 U/L, or the platelet count fell below 5 × 10^9^/L, the study drug was stopped. Adverse events (AEs) and details of severity and likely relatedness to the study drug were recorded in the case report form. Serious adverse events (SAEs) were reported to the relevant IRBs and to an independent data and safety monitoring committee (DSMB).

Hematocrit, platelet, and total cholesterol measurements were performed at enrollment (study day 1), then daily to study day 6, and at the follow-up visit 4 weeks after enrollment. Renal and liver function tests, electrolytes, and coagulation profiles were carried out on study days 1, 3, 5, and at follow-up. A 2-dimensional ultrasound scan was performed on illness day 6 to quantify the presence of plasma leak. Quality of life was measured using a visual analogue scale daily.

Serological and virological tests were used to confirm the dengue diagnosis and identify the infecting serotype, as outlined in Supplementary Text 1. Viremia was quantified on the daily plasma samples using a serotype-specific, real-time reverse transcription polymerase chain reaction assay [20].

The DSMB provided study oversight. Safety reviews were performed at the end of the first phase, and after the 30th and 100th patients were recruited in phase 2.

### Outcomes

This was an exploratory study focusing on safety, with the primary outcome defined as a comparison of the proportion of patients with any AE, and with any SAEs, between the treatment arms. The cutoffs described above for worsening hepatitis (ALT >250 U/L) and myositis (CK >1000 U/L) defined certain prespecified adverse events, but we also graded all laboratory abnormalities as grade 1–4 following the established Common Terminology Criteria for Adverse Events system. Secondary outcomes, as detailed in Supplementary Text 1, were as follows: (1) disease progression as defined by admission to intensive care unit, development of severe dengue (shock, severe bleeding, or neurological involvement), or death; (2) fever clearance time; (3) the area under the log_10_-transformed plasma viremia curve between days 3 and 6 of illness; and (4) the lowest quality of life score between days 2 and 6 of the study. Additional exploratory outcomes, intended primarily to evaluate vascular leakage severity and other characteristic features of dengue, are also described in Supplementary Text 1.

### Statistical Analysis

The planned sample size of 300 patients in phase 2 was based on medical and feasibility considerations as well as power considerations for key endpoints. In a previous dengue trial performed by our group, approximately 10% and 30% of participants experienced at least 1 SAE or AE, respectively [21]. The planned sample size guaranteed a power of 80% to detect an increase of 12% in the SAE frequency or 16% in the AE frequency. More extensive power considerations are discussed in the published protocol [19].

Cohort 1 was analyzed descriptively. The main analysis population included all patients in cohort 2 following the intention-to-treat principle, with the principal analyses defined a priori in a written analysis plan. The proportion of patients with AEs was compared between the treatment arms using Fisher exact test, while the predefined secondary endpoints were compared based on linear regression for continuous endpoints, logistic regression for binary endpoints, and Cox regression for time-to-event endpoints. The main explanatory variable in the regression models was the treatment assignment, and all analyses were adjusted for day of illness at recruitment. Comparisons of laboratory markers were additionally adjusted for the predose enrollment value, and also for serotype and immune status for plasma viremia comparisons.

All analyses were performed with the statistical software R version 3.1.3 (R Foundation for Statistical Computing, Vienna, Austria).

## RESULTS

Patients were recruited into phase 1 in November and December 2012 (the trial profile is shown in Supplementary Figure 1). The baseline characteristics were similar in the 2 study groups; illness duration and AE rates were also comparable, and all AEs resolved fully (Supplementary Tables 1 and 2).

**Figure 1.**
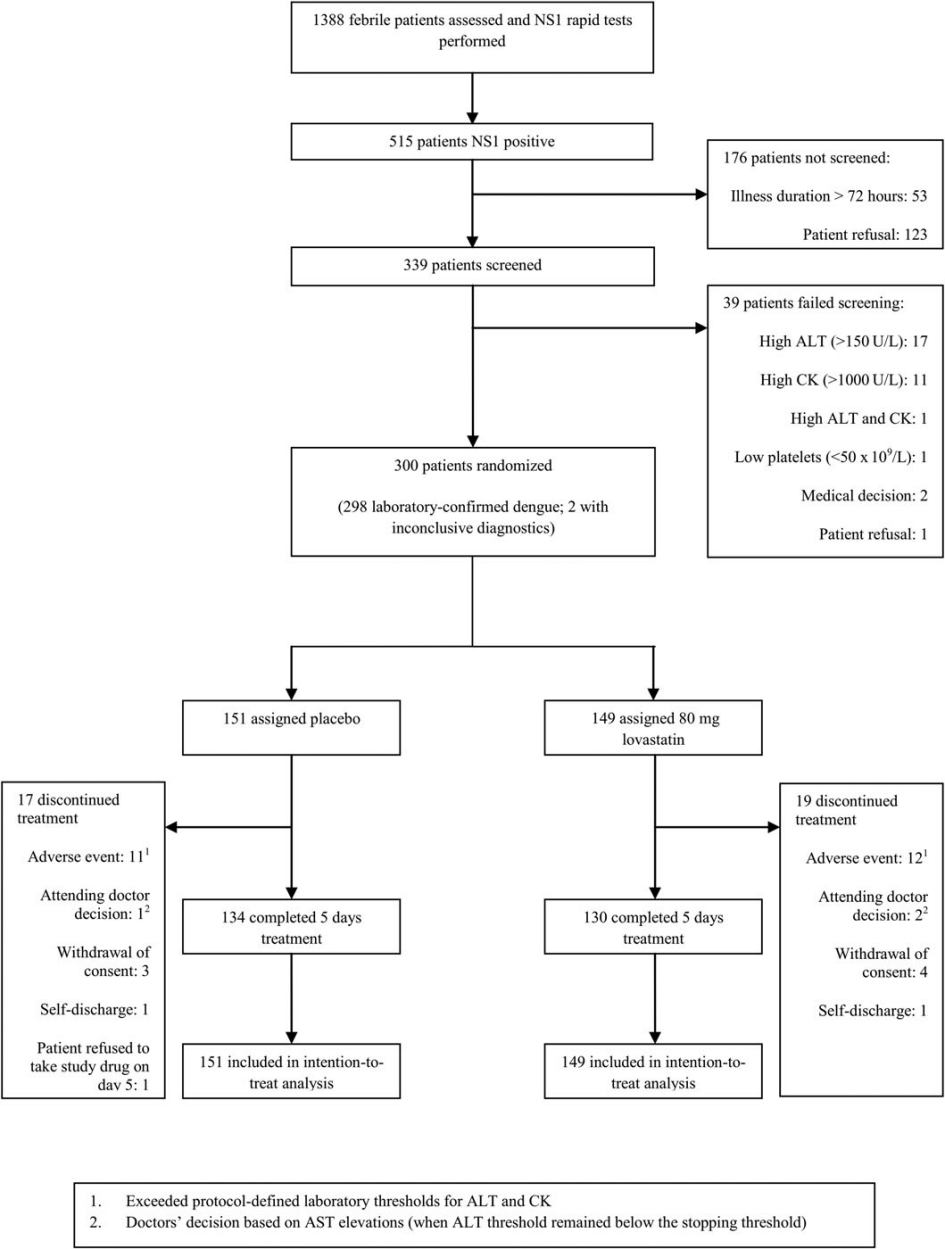
Study enrollment and follow-up for phase 2 of the study. Abbreviations: ALT, alanine aminotransferase; AST, aspartate aminotransferase; CK, creatine kinase; NS1, dengue nonstructural protein 1.

Following the initial safety review by the DSMB, phase 2 ran from April 2013 to January 2015. Figure 1 shows the trial profile. Of the 515 subjects with a positive NS1 test who were assessed for eligibility, 339 underwent formal screening and 300 patients were enrolled and randomized.

The baseline characteristics were similar between the 2 study groups (Table 1). Treatment was stopped prematurely in 17 placebo and 19 lovastatin recipients. Eleven patients in the placebo arm developed laboratory abnormalities on study day 3 where the protocol mandated cessation of the study drug: 10 with ALT elevations >250 U/L and 1 with a CK elevation >1000 U/L. Twelve patients in the lovastatin arm had ALT elevations >250 U/L that required the drug to be stopped. These abnormalities were not associated with clinical deterioration. The study physicians elected to stop the study drug in 3 other cases (1 placebo, 2 lovastatin) due to aspartate aminotransferase elevations, although the ALT levels did not meet the stopping criteria.

**Table 1.** Baseline Characteristics of the Patients in Phase 2 of the Study

Characteristic	Placebo (n = 151)	80 mg Lovastatin (n = 149)
Age, y	27 (21–33)	25 (21–34)
Sex		
Male	66 (44%)	65 (44%)
Female	85 (56%)	84 (56%)
Temperature, °C	38.2 (37.7–38.5)	38.2 (37.7–38.5)
Hours from fever onset to first treatment dose^a^		
0–23	8 (5%)	6 (4%)
24–47	45 (30%)	35 (23%)
48–71	79 (52%)	80 (54%)
72–77	19 (13%)	28 (19%)
DENV serotype		
1	62 (41%)	53 (36%)
2	18 (12%)	15 (10%)
3	13 (9%)	15 (10%)
4	52 (34%)	64 (43%)
Negative	6 (4%)	2 (1%)
Immune status		
Probable primary	35 (23%)	43 (29%)
Probable secondary	111 (74%)	96 (64%)
Inconclusive	5 (3%)	10 (7%)
Plasma viremia, log_10_ copies/mL	7.9 (7.1–8.9)	7.8 (6.9–8.6)
Hematocrit, %	41.8 (39.2–44.8)	41.0 (38.7–44.2)
Platelet count, ×10^9^ cells/L	111 (90–140)	112 (89–149)
WBC, ×10^6^/dL	3.2 (2.3–4.0)	3.1 (2.3–3.9)
AST, U/L	51 (36–79)	45 (32–72)
ALT, U/L	38 (25–67)	31 (23–60)
CK, U/L	95 (67–153)	92 (67–123)
Cholesterol, mmol/L	3.8 (3.3–4.3)	3.8 (3.2–4.3)

The summary statistic is the absolute count (%) for categorical variables and median (interquartile range) for continuous variables. Data were complete except for 1 missing CK and 1 missing cholesterol value in the lovastatin group, and 3 missing viremia at baseline in the placebo group. Moreover, there were 8 patients with negative plasma viremia at baseline, which were excluded from the viremia summary. Of note, dengue virus serotype could be determined in 1 of these cases, based on subsequent plasma viremia measurements.

Abbreviations: ALT, alanine aminotransferase; AST, aspartate aminotransferase; CK, creatine kinase; DENV, dengue virus; WBC, white blood cell count.

^a^ All patients presented within 72 hours of fever onset. Screening was completed within 4 hours (as per protocol) in all except 2 and 5 cases in the placebo and lovastatin arms, respectively. In these cases, the first dose of the study drug was given after 5 hours (77 hours from fever onset).

Treatment allocation was unblinded in 1 case (placebo arm), where a pregnancy test requested by the patient on study day 6 was positive (the enrollment test was negative). Two patients in the lovastatin arm withdrew from the study within 24 hours (1 self-discharged and 1 withdrew consent after the first dose of study drug); no AEs were documented in these cases. Data from other participants who withdrew from the study later are included in the analyses up to the time of withdrawal.

The number of AEs did not differ significantly between the groups (Table 2). Ninety-seven of 151 (64%) patients in the placebo group and 82 of 149 (55%) patients in the lovastatin group had a clinical and/or prespecified laboratory AE (*P* = .13). Eight patients in the placebo group and 4 patients in the lovastatin group experienced an SAE (*P* = .38). One hundred twelve of 151 (74%) patients in the placebo group and 110 of 149 (75%) patients in the lovastatin group had at least 1 grade 3 or 4 laboratory AE identified (*P* = 1).

**Table 2.** Primary Outcomes: Adverse Event Details

Adverse Event	Placebo (n = 151)	80 mg Lovastatin (n = 149)	*P* Value
Clinical and/or prespecified laboratory AEs			
Any	97 (64%)	82 (55%)	.13
Abdominal pain	4 (3%)	5 (3%)	.75
Diarrhea	39 (26%)	29 (19%)	.22
Vomiting	27 (18%)	22 (15%)	.53
Bleeding^a^	51 (34%)	38 (26%)	.13
Muscle pain	7 (5%)	7 (5%)	1
Hepatitis^b^	14 (9%)	16 (11%)	.70
Myositis^c^	1 (1%)	1 (1%)	1
Thrombocytopenia^d^	1 (1%)	1 (1%)	1
Serious AEs			
Any	8 (5%)	4 (3%)	.38
Diarrhea	1 (1%)	0	1
Hepatitis^b^	6 (4%)	3 (2%)	.50
Thrombocytopenia^d^	1 (1%)	1 (1%)	1
Grade 3/4 laboratory AEs			
Any	112 (74%)	110 (74%)	1
High ALT (>200 U/L [M] or 185 U/L [F])	65 (43%)	65 (44%)	1
High AST (>200 U/L [M] or 185 U/L [F])	65 (43%)	65 (44%)	1
High creatine kinase (>570 U/L)	9 (6%)	6 (4%)	.60
Low platelet count (<50 × 10^9^/L)	89 (59%)	84 (56%)	.73
Low sodium (<120 mmol/L)	1 (1%)	0	1
Low white cell count (<1 × 10^9^/L)	1 (1%)	3 (2%)	.37

Data are presented as No. (%) unless otherwise specified. *P* values derived from Fisher exact test.

Abbreviations: AE, adverse event; ALT, alanine aminotransferase; AST, aspartate aminotransferase.

^a^ Mild mucosal bleeding (eg, gum, nose, vaginal bleeding) in all cases. In 1 patient the attending physician elected to administer a platelet transfusion as the platelet count was 15 × 10^9^/L although the bleeding was not clinically severe.

^b^ Hepatitis defined here as ALT >250 U/L.

^c^ Myositis defined here as creatine kinase >1000 U/L.

^d^ Thrombocytopenia defined as a platelet count that led to clinical concern. In these cases the platelet nadir fell to <20 × 10^9^/L and was slow to recover. Conventionally, in Vietnam patients are kept in hospital until the platelet count is >50 × 10^9^/L.

Biochemical evidence for hepatic dysfunction was common, with similar proportions of participants (31/151 [21%] and 38/149 [26%]) in the placebo and lovastatin groups, respectively, exceeding the prespecified ALT cutoff of 250 U/L at some time during the acute illness, in most cases after completion of study drug (Supplementary Figure 3). Although thrombocytopenia was common, in no case did the platelet count drop to <5 × 10^9^/L; however, in 1 patient in each group the platelet recovery was slow, requiring inpatient monitoring for an additional 48 hours. Minor mucosal bleeding was also frequent in both treatment arms. No participant in phase 2 experienced significant mucosal bleeding, but in 1 case the attending physician elected to give a platelet transfusion, as the count was 15 × 10^9^/L when bleeding occurred. One patient in each group developed clinical myositis with a CK >1000 U/L, although less marked CK elevations were common.

The SAEs were all in the category of “prolonged hospitalization.” In 11 patients these prolonged hospital stays were for monitoring of abnormal laboratory tests (9 patients had hepatitis, plus the 2 cases with thrombocytopenia mentioned above) without clinical symptoms, while in 1 placebo recipient the prolonged stay was for diarrhea and persistent fever. All SAEs resolved fully.

**Figure 2.**
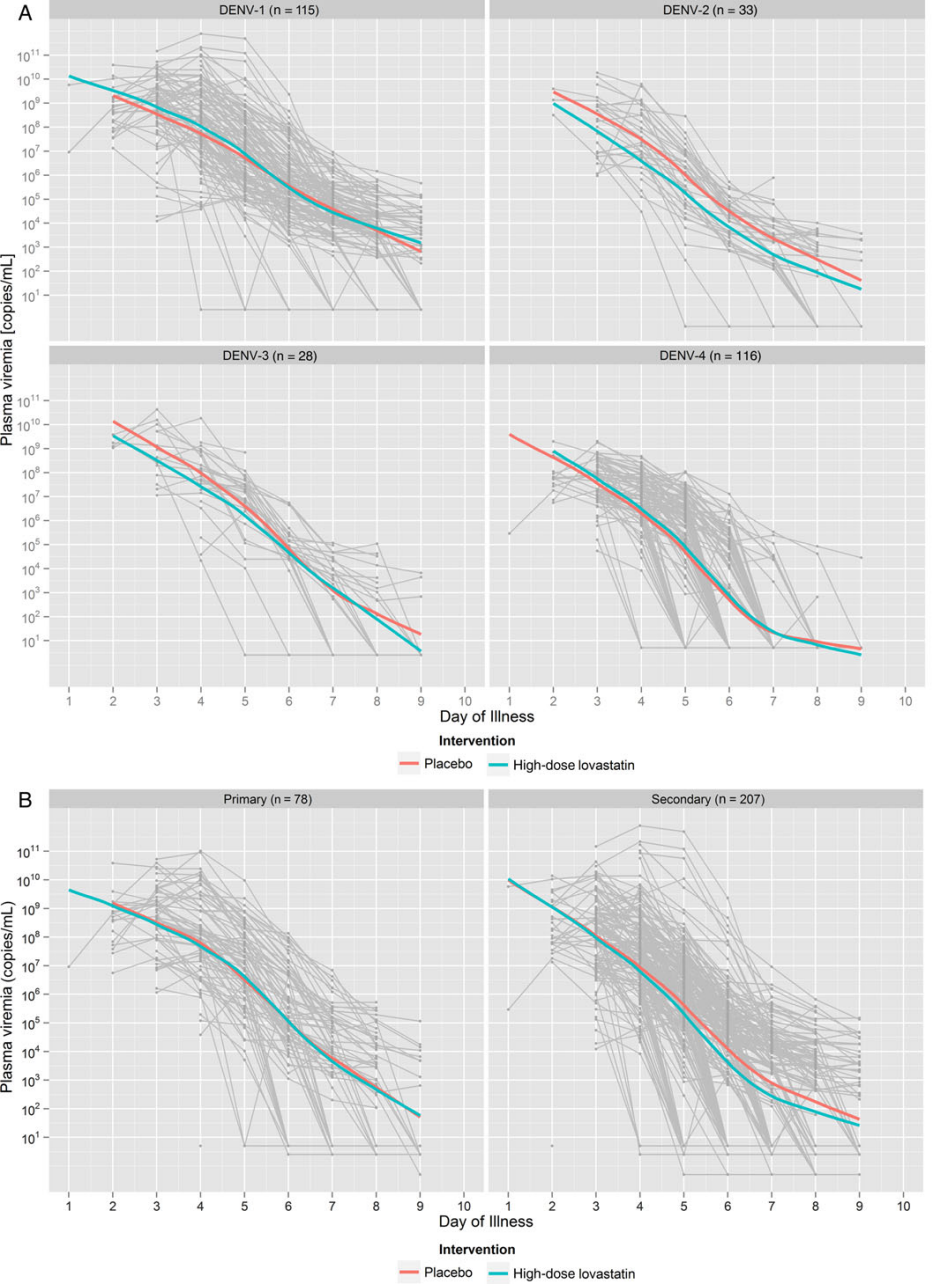
Viremia levels in lovastatin- and placebo-treated patients. Viremia is shown by serotype (*A*) and immune status (*B*). Values below the detection limit were imputed by half of the serotype-specific detection limits. The colored lines in each graph correspond to loess scatterplot smoothers derived from local polynomial regression fitting to data from each treatment arm. The gray background lines represent individual patient data. Overall, viremia kinetics did not differ significantly between the treatment groups. Abbreviation: DENV, dengue virus.

Two patients in the placebo group developed hypovolemic shock, and 1 patient in the lovastatin group was admitted to intensive care for close monitoring. The fever clearance time did not differ significantly between the study groups (Table 3 and Supplementary Figure 2). Lovastatin had no observable effect on dengue viremia kinetics overall, or on the quality of life scores (Table 3). Post hoc subgroup analyses to investigate potential effects by serotype and immune status suggested a possible benefit of lovastatin in dengue virus (DENV) type 2–infected participants for viremia AUC, and in secondary infection for time to undetectable viremia (Figure 2 and Supplementary Table 3). However, the corresponding overall tests for treatment effect heterogeneity by serotype or immune status did not reach significance, and these subgroup analyses were not predefined. Thus, these results should be interpreted with caution [22].

**Table 3.** Secondary Outcomes

Outcome	Placebo (n = 151)	80 mg Lovastatin (n = 149)	Comparison Estimate (95% CI); *P* Value
Disease progression			OR of having disease progression:
Frequency, %	2/151 (1%)	1/147 (<1%)	0.53 (.02–5·57); *P* = .59
Time to fever clearance, d			HR of time to fever clearance:
Median (IQR)	4 (3–5)	4 (3–5)	0.93 (.72–1.21); *P* = .60
AUC of viremia between days 3 and 6 of illness, log_10_ copies/mL × number of days			
Median (IQR)	16.8 (12.5–21.6)	16.1 (11.5–20.2)	Adjusted absolute mean difference:
Mean	16.9	16.2	−0.2 (−.9 to .5); *P* = .56
Minimum quality of life score between days 2 and 6 of study			
Median (IQR)	50 (40–60)	50 (50–60)	Adjusted absolute mean difference:
Mean	51	54	1 (−1 to 3); *P* = .38

Median (IQR) for time to fever clearance was based on Kaplan–Meier estimation.

Outcomes of 2 patients in the lovastatin arm were not available due to early withdrawal. In addition, 3 patients (1 in the lovastatin arm) were excluded from the analysis of fever clearance due to having already defervesced at baseline; 11 patients (2 in the lovastatin arm) were excluded from the analysis of viremia as the initial dengue virus reverse transcription polymerase chain reaction was negative or missing; and 4 patients (2 in the lovastatin arm) were excluded from the analysis of quality of life due to missing data.

Abbreviations: AUC, area under the log_10_-transformed plasma viremia curve; CI, confidence interval; HR, hazard ratio; IQR, interquartile range; OR, odds ratio.

There were no significant differences between the treatment arms in the various predefined exploratory endpoints (Table 4), except in the peak percentage change in total cholesterol levels relative to enrollment values; a greater relative reduction was observed in the lovastatin group (30%) compared with the placebo group (23%) (*P* < .0001; Supplementary Figure 4). Markers for plasma leakage were similar between the treatment arms.

**Table 4.** Exploratory Outcomes

Outcome	Placebo (n = 151)	80 mg Lovastatin (n = 149)	Comparison Estimate (95% CI); *P* Value
Platelet nadir (between days 3 and 8 of illness), × 10^9^ cells/L	43 (24–64)	44 (26–71)	2 (−4 to 9); *P* = .45
Peak hematocrit (between days 3 and 8 of illness), %	46.0 (42.8–49.3)	44.3 (42.0–49.0)	−0.6 (−1.2 to .1); *P* = .10
Peak percentage change in hematocrit from baseline (between days 3 and 8 of illness), %	9.7 (4.5–15.2)	8.9 (5.0–12.7)	−1.3 (−2.9 to .3); *P* = .11
Peak ALT (between day 3 and 8 of illness), U/L	110 (64–184)	89 (46–185)	6 (−28 to 40); *P* = .72
Peak ALT (during study), U/L	140 (82–222)	133 (67–269)	17 (−20 to 54); *P* = .37
Peak CK (between day 3 and 8 of illness), U/L	125 (80–199)	108 (70–200)	56 (−85 to 196); *P* = .44
Peak CK (during study), U/L	125 (80–199)	116 (72–200)	57 (−83 to 197); *P* = .43
Peak percentage change in cholesterol from baseline (between days 2 and 6 of study), %	−23.1 (−29.4 to −15.4)	−30.4 (−38.4 to −23.6)	−8.5 (−11.3 to −5.6); *P* ≤ .0001
Peak viremia (between days 2 and 6 of study), log_10_ copies/mL	7.5 (6.4–8.7)	7.4 (6.5–8.5)	0.0 (−.2 to .2); *P* = .95
AUC of viremia between days 1 and 6 of study,			
log_10_ copies/mL × days	26.2 (18.2–31.8)	22.7 (17.6–30.0)	−0.6 (−1.7 to .5); *P* = .31
Undetectable viremia achieved	71/145 (49%)	87/147 (61%)	1.37 (.98–1.91); *P* = .06
Ascites on ultrasound between days 5 and 7 of illness	23/142 (16%)	17/137 (12%)	0.69 (.35–1.37); *P* = .29
Pleural effusion on ultrasound between days 5 and 7 of illness	14/142 (10%)	11/137 (8%)	0.79 (.34–1.81); *P* = .58
Colloid requirement	1/151 (0.66%)	0/147 (0%)	NA
Total no. of days in hospital	8 (7–8)	8 (7–8)	0.0 (−.3 to .3); *P* = .89

Summary statistics are median (interquartile range) for continuous variables and frequency/No. (%) for categorical variables. The number of missing values was <8% in both arms for all outcomes.

Estimates are absolute mean differences (for all continuous laboratory endpoints), odds ratios of having the event (for binary endpoints), or hazard ratio of time to undetectable viremia (for undetectable viremia achieved). An estimate for colloid requirement was not available because there was only 1 case in the placebo group who required colloids.

All analyses were adjusted for day of illness at baseline. Analyses for continuous laboratory endpoints were also adjusted for the baseline value of the corresponding endpoint. Analyses for plasma viremia (peak viremia, AUC of viremia, time to undetectable viremia) were additionally adjusted for dengue serotype and immune status.

Abbreviations: ALT, alanine aminotransferase; AUC, area under the log_10_-transformed plasma viremia curve; CI, confidence interval; CK, creatine kinase; NA, not applicable.

## DISCUSSION

We hypothesized that the benefits associated with statin use in several observational studies of acute inflammatory syndromes might be relevant to dengue, a condition in which endothelial dysfunction is central to pathogenesis. In this randomized, double-blind, placebo-controlled trial in Vietnamese adults with dengue, we found that lovastatin was safe and well tolerated. Specifically, we found no evidence of AEs on hepatic or muscle dysfunction, both characteristic features of acute dengue as well as recognized complications of statin therapy. However, we also found no evidence of a beneficial effect on any clinical or virological endpoints.

Although our study did not include pharmacokinetic analysis, we used 80 mg lovastatin daily, and it is reasonable to assume that therapeutic concentrations were achieved as evidenced by the significantly greater reduction in cholesterol in the lovastatin group. The rates of clinical and laboratory AEs were similar between the treatment arms. Rates of SAEs were also similar, and in all cases these events were classified as serious due to prolonged hospitalization for laboratory monitoring rather than on the basis of clinical deterioration. Biochemical abnormalities were observed frequently in both treatment arms, and were no more prevalent in the lovastatin arm.

Progression to severe dengue occurred infrequently in the study population (1%) and there was no difference in the rate of disease progression between the study groups. In view of the small number of events, it is possible that the study missed a small beneficial effect. The frequency of dengue shock syndrome is higher in children and, hence, children are the preferred patient population for investigation of drugs with endothelial stabilizing properties [23]. However, since we observed no differences in the magnitude of hemoconcentration or in the presence of effusions on ultrasound between the study groups in this adult trial, we do not consider there to be a compelling case for a trial of statin therapy in children at present. We also found no other evidence of an anti-inflammatory effect; in particular, fever clearance times were unaffected by statin therapy.

Another limitation of our study is that we obtained sparse data on DENV-2 and DENV-3 due to the dominance of DENV-1 and DENV-4 during the study. Although in vitro laboratory studies have suggested that statins may reduce dengue virion assembly, we found no evidence of an antiviral effect [14]. It is possible that the effects of statins may differ between the serotypes and it is interesting to note that the work that suggested a potential antiviral effect used DENV-2 [14–16].

As in other recent trials of antiviral and immunomodulatory agents for dengue [21, 24–26], we administered the study drug within 72 hours of illness onset. It is possible that even initiating a therapeutic within this timeframe is too late to modulate the disease pathways. Peak viremia typically occurs earlier in the illness [27]. In addition, there is evidence that endothelial dysfunction is established by 72 hours [28]. To favorably modulate outcome, earlier intervention may be necessary, which is a significant hurdle in many endemic regions as patients rarely present to healthcare so early. It also raises the question of whether therapeutic development should focus more on identifying chemoprophylactic agents that could be used in those at most risk of severe disease, and also on optimizing supportive care for those who do develop severe disease. The ability to distinguish patients at risk of severe disease early in the course of illness could lead to a more targeted clinical trial approach. The use of dengue human infection models may also allow streamlining of potential therapeutic candidates [29].

Following the initial hope raised by observational studies suggesting that prior statin therapy might improve outcomes in a variety of inflammatory conditions, several prospective trials investigating statins as adjunctive therapy have recently been published [12, 13, 30]; similar to our study findings, none of these trials showed a beneficial role for de novo statin therapy. Despite these negative results, this “discovery-in-practice” approach remains important given the high preclinical failure rate observed with traditional drug discovery. A dengue therapeutic or chemoprophylactic agent remains a desirable component of a dengue control package and, given the ongoing expansion of dengue's geographic footprint, both drug development approaches need to be explored.

In conclusion, we have shown that lovastatin therapy is safe in adult patients with dengue, although we did not demonstrate a clinical or virological benefit. Although the study findings do not endorse adjunctive statin therapy for dengue, the data provide reassurance to clinicians about the safety of continuing statin therapy in patients who develop dengue. Early diagnosis and recognition of severe features, together with good supportive care, remain central to effective clinical management.

## Supplementary Data

Supplementary materials are available at (http://cid.oxfordjournals.org). Consisting of data provided by the author to benefit the reader, the posted materials are not copyedited and are the sole responsibility of the author, so questions or comments should be addressed to the author.

Supplementary Data
