# Supplementary material for: Lovastatin for the Treatment of Adult Patients With Dengue: A Randomized, Double-Blind, Placebo-Controlled Trial
Source: Clin Infect Dis. 2015 Nov 12;62(4):468–76. doi: 10.1093/cid/civ949 (PMC4725386; doi:10.1093/cid/civ949)
Supplement: Supplementary Data [file supp_civ949_civ949supp.docx]

**Definitions used in Lovastatin for Dengue Trial**

**Clinical Definitions**

SHOCK: Cardiovascular decompensation (indicated by tachycardia, cool peripheries and narrowing of the pulse pressure to less than 20 mmHg) requiring fluid resuscitation and thought to be due to plasma leak.

SEVERE BLEEDING: Bleeding was defined as clinically severe if it resulted in haemodynamic instability and required fluid resuscitation or a blood transfusion. In addition, any bleed that required an intervention to control the bleeding (e.g. nasal packing) was classified as severe. Any intracranial bleed or bleed that resulted in death were classified as severe.

CNS INVOLVEMENT: This was defined as any alteration of consciousness (GCS<15), or occurrence of convulsions or any focal neurological deficit even if transient.

DISEASE PROGRESSION: This was present if any of the following occurred: (1) transfer to ICU, (2) shock, (3) severe bleeding, (4) CNS involvement, or (5) death.

FEVER CLEARANCE TIME: This was defined as the time (days) from enrolment to the first time the temperature fell to < 37.5 ^o^C and remained below this level for 24 hours, or less than 24 hours if the patient was well enough to be discharged home.

DAY OF ILLNESS: This was calculated using the date of illness onset and the date of enrolment. This definition meant that some patients who were within 72 hours of fever onset were actually on day four of illness at enrolment.

**Laboratory Definitions**

DENGUE DIAGNOSIS: Serological responses were detected using immunoglobulin M (IgM) and immunoglobulin G (IgG) antibody-capture enzyme-linked immunosorbent assays (Panbio, Australia). DENV plasma viremia levels were measured by a validated quantitative RT-PCR assay. A patient was deemed to have laboratory-confirmed dengue if DENV was detected using the RT-PCR assay or if there was evidence of IgG or IgM seroconversion in paired samples.

All patients had a positive NS1 rapid test at screening. If a patient had a negative RT-PCR result and inconclusive serology they were deemed to have an inconclusive laboratory diagnosis – this occurred in two patients (both in the placebo arm). Eight patients (two in the lovastatin arm) had negative RT-PCR results and three patients (all in the placebo arm) did not have baseline viremia – these patients were excluded from the analysis of viremia kinetics.

DENGUE IMMUNE STATUS: We classified serological profiles as "probable secondary" when >22 Units of IgG (Panbio’s recommended cut-off for a positive result) were detected in either acute or early convalescent samples. If acute and early convalescent samples were IgM positive but IgG negative then we classified as "probable primary". Where IgM or IgG tests results were equivocal or inconsistent we classified the serological profile as "indeterminate”.

LABORATORY ADVERSE EVENTS: In addition to the pre-specified cut-offs for particular parameters (ALT, CK and platelet count) laboratory adverse events were also graded according to the values in the following table, following the convention of the CTCAE guidelines.^1^

|  | **Grade 1** | **Grade 2** | **Grade 3** | **Grade 4** |
| --- | --- | --- | --- | --- |
| **Haematological** |  |  |  |  |
| Haemoglobin | 10g/dl – 12.5g/dl | 8 – 9.9 g/dl | 6.5 –7.9g/dl | <6.5 g/dl |
| White cell count | 2 – 2.94 K/μl | 1.0 - 1.99 K/μl | <1.0 K/μl |  |
| Platelets | 75 – 200 K/μl | 50 – 74 K/μl | 20 - 49 K/μl | <20 K/μl |
| **Biochemical** |  |  |  |  |
| Sodium (low) | 130 – 135 mmol/L | 120 - 129 mmol/l | <120 mmol/l | - |
| Sodium (high) | 146 – 150 mmol/L | 151 – 155 mmol/L | 156 – 160 mmol/l | >160 mmol/l |
| Potassium (low) | 3.0 – 3.5 mmol/l | 2.5 – 2.9 mmol/l | <2.5 mmol/l |  |
| Potassium (high) | 5 – 5.5 mmol/l | 5.6 – 6.0 mmol/l | 6.1 – 7.0 mmol/l | >7.0 mmol/l |
| Creatinine | Male 121 – 180 µmol/L  Female 101 – 150 µmol/L | Male 181 – 360 µmol/L  Female 151 – 300 µmol/L | Male > 360 µmol/L  Female > 300 µmol/L | - |
| Total Bilirubin | 18 – 27 µmol/L | 28 – 51 µmol/L | 52 – 170 µmol/L | > 170 µmol/L |
| AST | Male 41 – 120 U/L  Female 38 – 111 U/L | Male 121 – 200 U/L  Female 112 – 185 U/L | Male 201 – 400 U/L  Female 186 – 370 U/L | Male > 400 U/L  Female >370 U/L |
| ALT | Male 41 – 120 U/L  Female 38 – 111 U/L | Male 121 – 200 U/L  Female 112 – 185 U/L | Male 201 – 400 U/L  Female 186 – 370 U/L^1^ | Male > 400 U/L  Female > 370 U/L |
| Creatine kinase | 191 – 285 U/L | 286 – 570 U/L | >570 U/L | - |
| Cholesterol | 5.21 – 7.75 mmol/L | 7.76 – 10.34 mmol/L | > 10.34 mmol/L | - |
| Prothrombin time | 13.4 – 20 seconds | 20.1 – 33 seconds | >33 seconds | - |

AREA UNDER THE LOG_10_-TRANFORMED PLASMA VIREMIA CURVE (AUC): The AUC was calculated based on the recorded log_10_-transformed plasma viremia measurements using the trapezoidal rule. Intermittant missing viremia measurements were imputed using linear interpolation and missing values after the last non-missing measurement were imputed using the last observation carried forward. Values below the detection limit (“negative” measurements) were replaced by half of the detection limit before computation. For DENV-1 the detection limit is 5 copies per RT-PCR, for DENV-2 1 copy/RT-PCR, for DENV-3 5 copies/RT-PCR, and for DENV-4 10 copies/RT-PCR.^2^

**Exploratory endpoints**

The exploratory outcomes included the following laboratory values observed between days three and eight of illness (i.e. during the time the study protocol required a specific schedule of investigations, when dengue complications typically occur): platelet nadir, maximum haematocrit, peak percentage increase in haematocrit from baseline, and maximum ALT and CK. We also assessed the maximum recorded ALT and CK observed during the whole illness episode, recognising that some laboratory derangements occur later in the evolution of dengue, and the peak percentage decrease in cholesterol during follow-up from baseline.

The numbers of patients requiring colloid therapy, and the total number of days in hospital, were also compared between the groups.

Additional pre-specified outcomes were the time from enrolment to the first undetectable viremia (and the proportion of patients who achieved an undetectable viremia), the peak viremia and the log_10_-transformed plasma viremia AUC during in-patient follow-up.

**References**

1. US Department of Health and Human Services. Common Terminology Criteria for Adverse Events (CTCAE) Version 4.0. National Institutes of Health; National Cancer Institute; 2009.

2. Hue KD, Tuan TV, Thi HT, et al. Validation of an internally controlled one-step real-time multiplex RT-PCR assay for the detection and quantitation of dengue virus RNA in plasma. *J Virol Methods* 2011; **177**(2): 168-73.
