# Supplementary material for: Lovastatin for the Treatment of Adult Patients With Dengue: A Randomized, Double-Blind, Placebo-Controlled Trial
Source: Clin Infect Dis. 2015 Nov 12;62(4):468–76. doi: 10.1093/cid/civ949 (PMC4725386; doi:10.1093/cid/civ949)
Supplement: Supplementary Data [file supp_civ949_civ949supp_fig_legends.docx]

**Supplementary Figure 1:** Study enrolment and follow-up for phase one of the study.

**Supplementary Figure 2:** Kaplan-Meier of fever clearance time in lovastatin and placebo-treated patients. The proportions of patients who remained febrile during inpatient monitoring (study days one to five) are shown. There was no significant difference in fever clearance time between the treatment groups

**Supplementary Figure 3:** Evolution of hepatic enzyme levels. Scatterplot of AST and ALT levels at enrolment (study day 1) plotted against the peak values measured during the illness episode for all particpants in phase two of the study. Data for the placebo group, lovastatin group, and all patients are shown for AST (top 3 panels) and ALT (bottom 3 panels).

**Supplementary Figure 4:** Cholesterol levels by day of illness and treatment arm. The coloured lines in each graph correspond to loess scatterplot smoothers derived from local polynomial regression fitting to data from each treatment arm. The grey background lines represent individual patient data.
