# Supplementary figures and images for: Lovastatin for the Treatment of Adult Patients With Dengue: A Randomized, Double-Blind, Placebo-Controlled Trial
Source: Clin Infect Dis. 2015 Nov 12;62(4):468–76. doi: 10.1093/cid/civ949 (PMC4725386; doi:10.1093/cid/civ949)

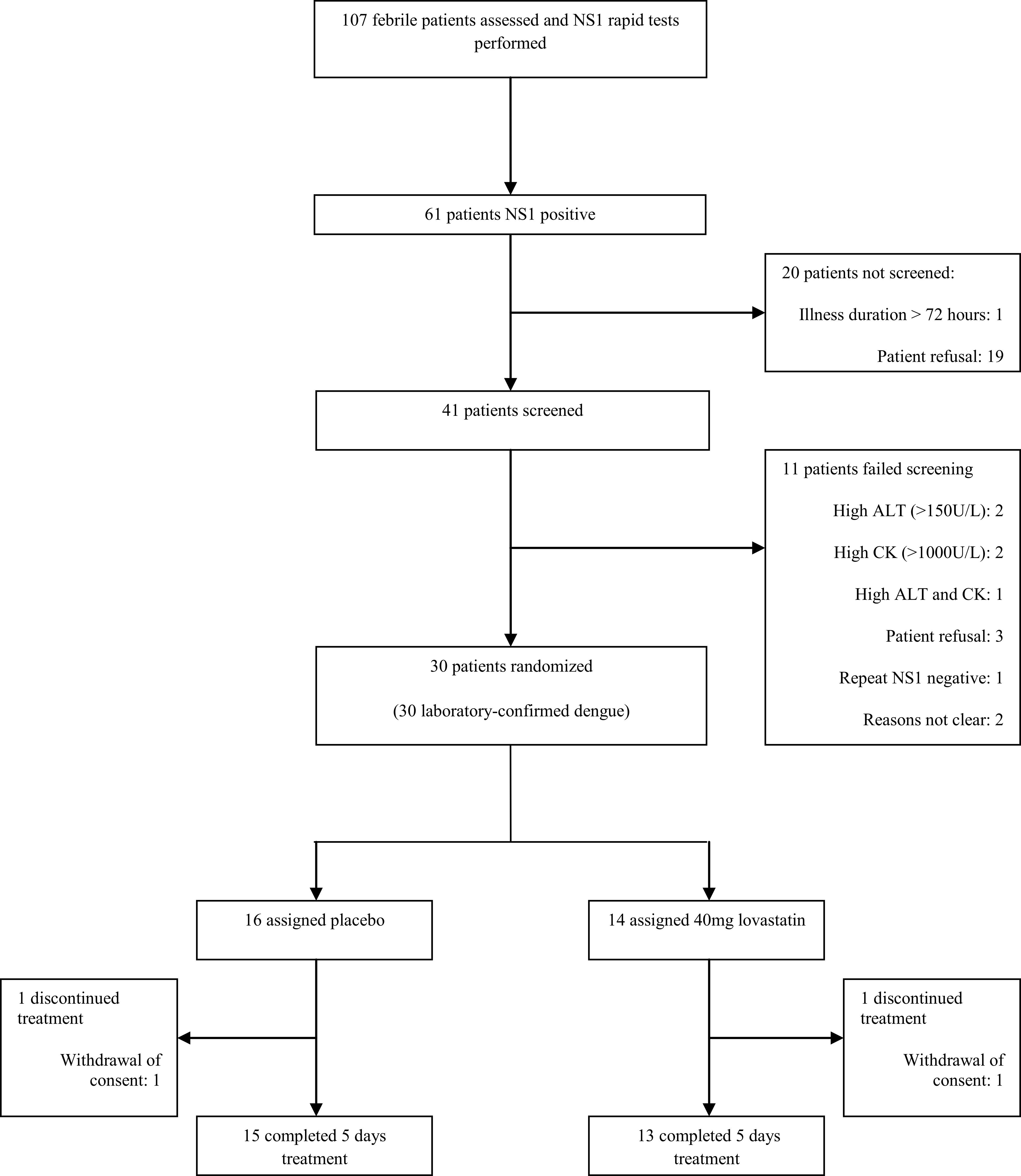

Supplement: Supplementary Data [file supp_civ949_civ949supp_fig1.tif]

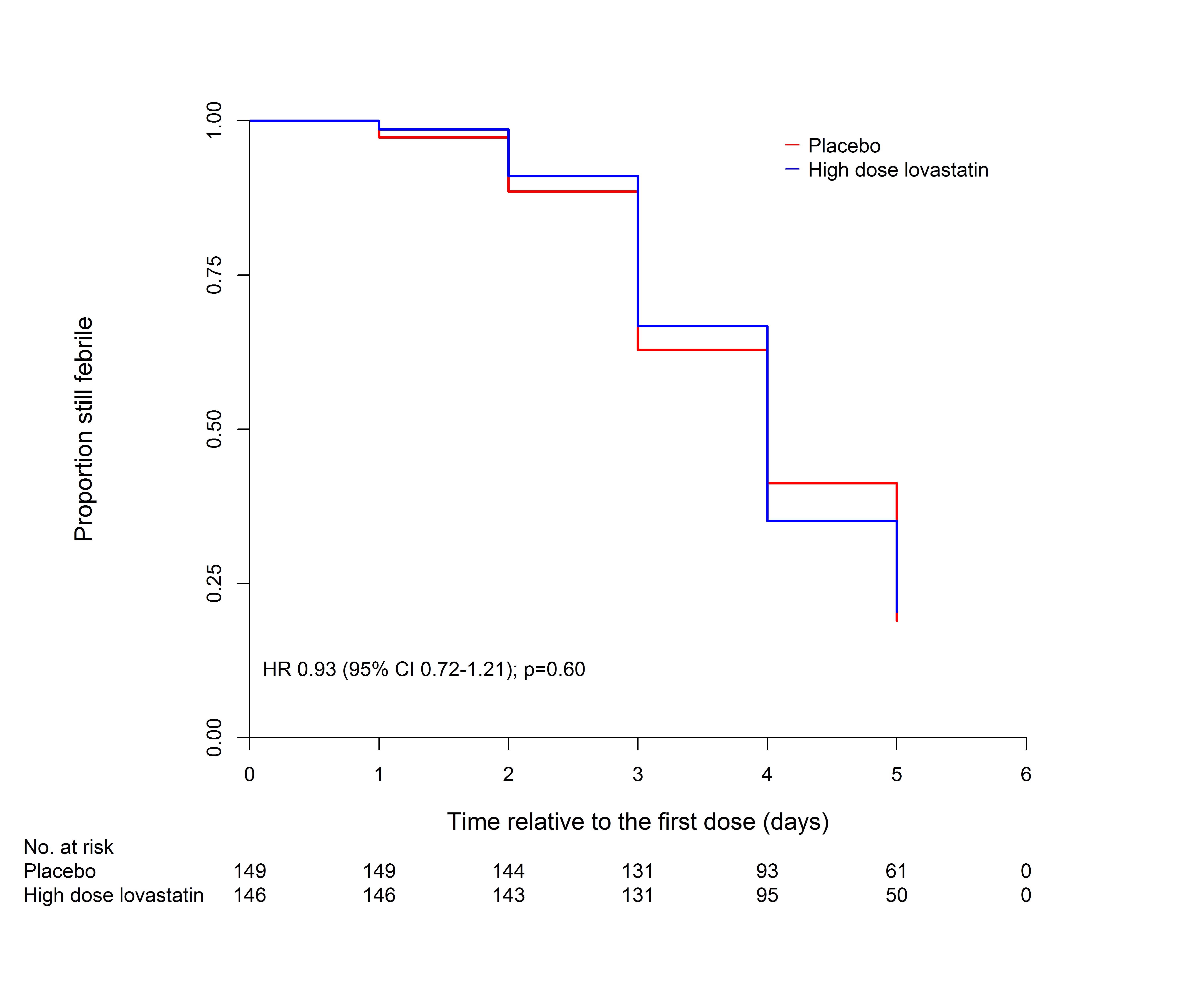

Supplement: Supplementary Data [file supp_civ949_civ949supp_fig2.tif]

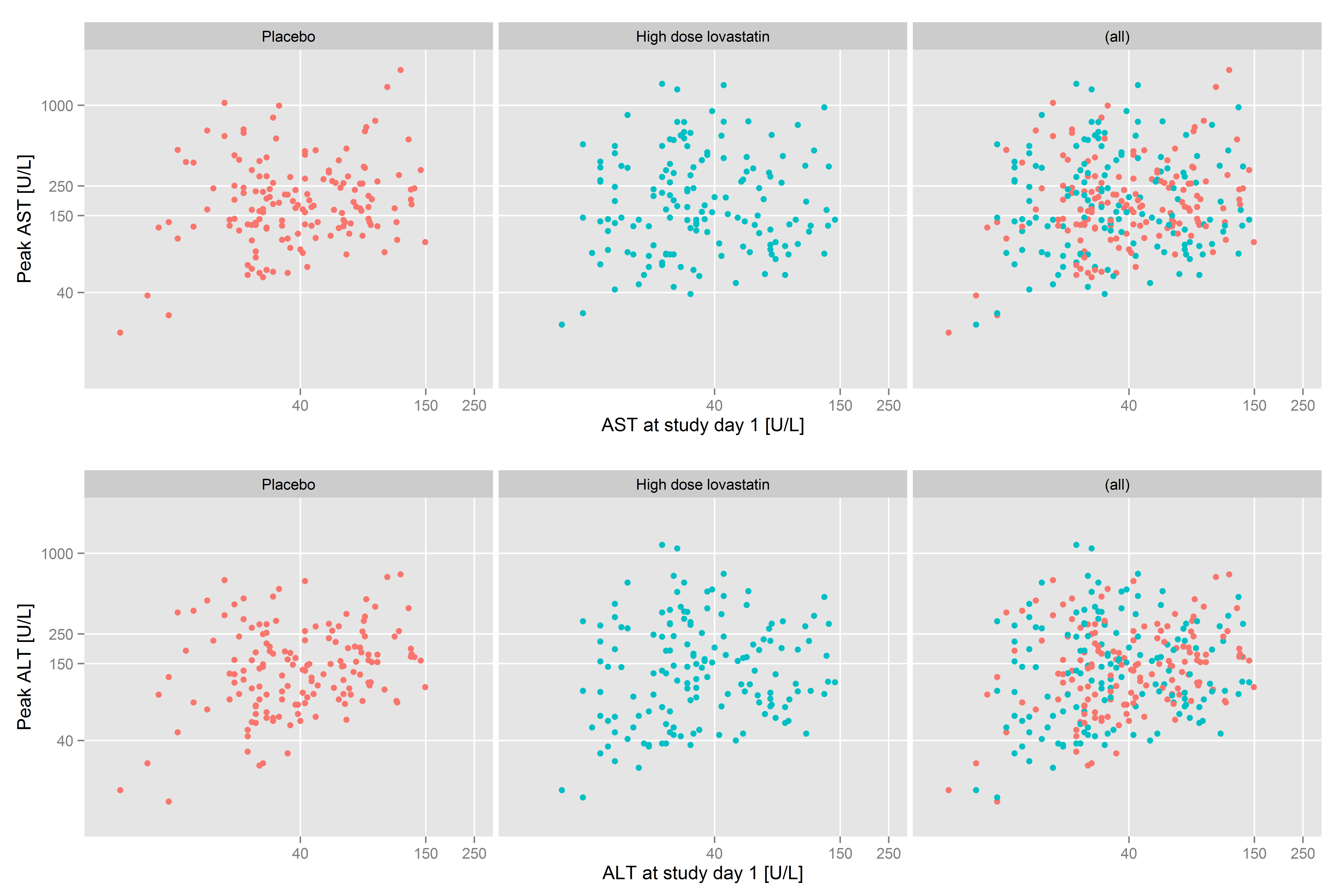

Supplement: Supplementary Data [file supp_civ949_civ949supp_fig3.tif]

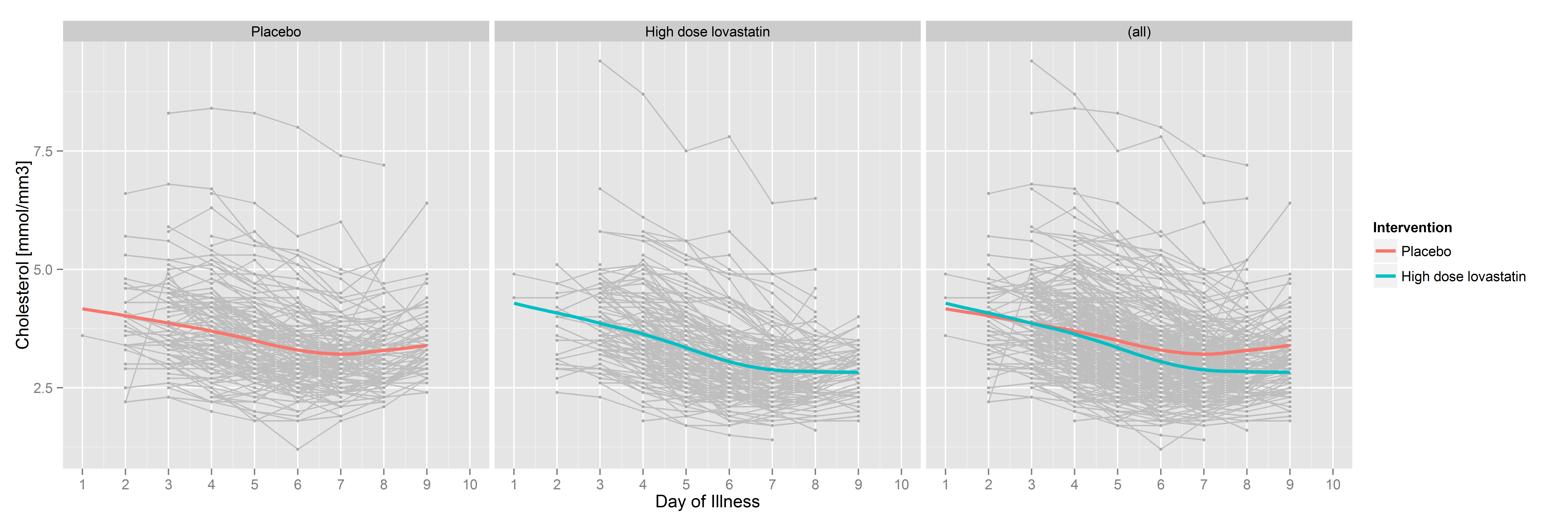

Supplement: Supplementary Data [file supp_civ949_civ949supp_fig4.tif]
