# Supplementary material for: Lovastatin for the Treatment of Adult Patients With Dengue: A Randomized, Double-Blind, Placebo-Controlled Trial
Source: Clin Infect Dis. 2015 Nov 12;62(4):468–76. doi: 10.1093/cid/civ949 (PMC4725386; doi:10.1093/cid/civ949)
Supplement: Supplementary Data [file supp_civ949_civ949supp_table1.docx]

|  | Placebo (n=16) | | | 40mg Lovastatin (n=14) | | |
| --- | --- | --- | --- | --- | --- | --- |
| Age (years) | 23 (19, 28) | | | 26 (21, 48) | | |
| Sex |  |  |  |  |  |  |
| Male | 8 (50%) | | | 5 (36%) | | |
| Female | 8 (50%) | | | 9 (64%) | | |
| Temperature (°C) | 39 (38·2, 39·5) | | | 39 (38·6, 39·6) | | |
| Hours from fever onset to first treatment dose^1^ |  |  |  |  |  |  |
| 0-23 | 0 | | | 0 | | |
| 24-47 | 8 (53%) | | | 6 (43%) | | |
| 48-71 | 7 (47%) | | | 7 (50%) | | |
| 72-85 | 0 | | | 1 (7%) | | |
| DENV serotype |  |  |  |  |  |  |
| 1 | 7 (44%) | | | 5 (36%) | | |
| 2 | 2 (12%) | | | 2 (14%) | | |
| 3 | 4 (25%) | | | 3 (21%) | | |
| 4 | 3 (19%) | | | 4 (29%) | | |
| Immune status |  |  |  |  |  |  |
| Probable primary | 3 (19%) | | | 0 | | |
| Probable secondary | 12 (75%) | | | 12 (86%) | | |
| Inconclusive | 1 (6%) | | | 2 (14%) | | |
| Plasma viraemia (log_10_ copies/mL) | 8·6 (7·6, 9·3) | | | 8.4 (7·8, 9·1) | | |
| Haematocrit (%) | 40·4 (37·9, 42·7) | | | 42·7 (40·0, 45·0) | | |
| Platelet count (x10^9^/L) | 130 (108, 171) | | | 106 (87, 147) | | |
| WBC (x10^6^/dL) | 3·7 (3·4, 4·5) | | | 3·2 (2·8, 4·1) | | |
| AST (U/L) | 35 (26, 61) | | | 39 (35, 90) | | |
| ALT (U/L) | 23 (16, 47) | | | 27 (22, 71) | | |
| CK (U/L) | 78 (66, 116) | | | 120 (91, 156) | | |

**Supplementary Table 1: Baseline characteristics of patients in phase one of the study**

The summary statistic is the absolute count (%) for categorical variables and the median (inter-quartile range) for continuous variables

1. Time of fever onset was missing in 1 patient in the placebo group who received the first dose of drug 1 day after the date of fever onset.
