# Supplementary material for: Lovastatin for the Treatment of Adult Patients With Dengue: A Randomized, Double-Blind, Placebo-Controlled Trial
Source: Clin Infect Dis. 2015 Nov 12;62(4):468–76. doi: 10.1093/cid/civ949 (PMC4725386; doi:10.1093/cid/civ949)
Supplement: Supplementary Data [file supp_civ949_civ949supp_table2.docx]

| Characteristic | Placebo (n=16) | | | 40mg Lovastatin (n=14) | | |
| --- | --- | --- | --- | --- | --- | --- |
| Any adverse event |  |  |  |  |  |  |
| Yes | 9 (56%) | | | 6 (43%) | | |
| No | 7 (44%) | | | 8 (57%) | | |
| Any serious adverse event^1^ |  |  |  |  |  |  |
| Any | 3 (19%) | | | 2 (14%) | | |
| Mucosal bleeding | 1 (6%) | | | 1 (7%) | | |
| Diarrhoea | 0 | | | 1 (7%) | | |
| Hepatitis | 1 (6%) | | | 0 | | |
| Urinary tract infection | 1 (6%) | | | 0 | | |

**Supplementary Table 2: Details of adverse events in phase one of the study**

The summary statistic is the absolute count (%).

1. All five serious adverse events were “prolonged hospitalisation”. These included two patients with mucosal bleeding (one in each treatment arm), one patient with diarrhoea (in the lovastatin arm), one patient with hepatitis (in the placebo arm), and one patient who developed a urinary tract infection (in the placebo arm).
