# Supplementary material for: Lovastatin for the Treatment of Adult Patients With Dengue: A Randomized, Double-Blind, Placebo-Controlled Trial
Source: Clin Infect Dis. 2015 Nov 12;62(4):468–76. doi: 10.1093/cid/civ949 (PMC4725386; doi:10.1093/cid/civ949)
Supplement: Supplementary Data [file supp_civ949_civ949supp_table3.docx]

Supplementary Table 3A. Post-hoc subgroup analysis of AUC log10 viremia between days 3 & 6 of illness by serotype and serology.

| **Subgroup** |  | **Placebo** |  |  | **80mg Lovastatin** | **Adjusted absolute mean** | **p value** | **p value for** |
| --- | --- | --- | --- | --- | --- | --- | --- | --- |
|  | **n** | **Median (IQR)** |  | **n** | **Median (IQR)** | **difference (95%CI)** |  | **heterogeneity** |
| Serotype |  |  |  |  |  |  |  | 0.54 |
| - DENV-1 | 62 | 20.0 (13.1, 23.9) |  | 53 | 18.8 (14.0, 23.2) | 0.0 (-1.1, 1.1) | 0.99 |  |
| - DENV-2 | 16 | 21.2 (16.3, 22.6) |  | 15 | 16.1 (11.5, 18.7) | -2.0 (-3.9, -0.1) | 0.04 |  |
| - DENV-3 | 12 | 14.5 (13.7, 21.3) |  | 15 | 19.7 (13.2, 23.2) | -1.5 (-4.4, 1.5) | 0.31 |  |
| - DENV-4 | 52 | 13.3 (10.3, 17.9) |  | 62 | 14.2 (10.2, 17.7) | 0.0 (-1.1, 1.2) | 0.96 |  |
| Serology |  |  |  |  |  |  |  | 0.39 |
| - Primary | 32 | 20.5 (14.3, 23.9) |  | 43 | 18.8 (15.8, 23.3) | 0.7 (-0.5, 1.8) | 0.25 |  |
| - Secondary | 107 | 15.5 (11.9, 21.2) |  | 94 | 14.1 (10.8, 18.7) | -0.6 (-1.5, 0.3) | 0.21 |  |

These analyses were based on linear regression with adjustment for day of illness at enrolment, indication of being enrolled before day 4 of illness, baseline log10 viremia, serotype and serology. Heterogeneity was assessed with a F-test for an interaction term between treatment group and sub-grouping variable in the statistical model.

Supplementary Table 3B. Post-hoc subgroup analysis of time to undetectable viremia for serotype and serology

| **Subgroup** |  | **Placebo** |  |  | **80mg Lovastatin** | **Hazard ratio of time to** | **p value** | **p value for** |
| --- | --- | --- | --- | --- | --- | --- | --- | --- |
|  | **Events/n** | **Median (IQR)** |  | **Events/n** | **Median (IQR)** | **undetectable viremia (95%CI)** |  | **heterogeneity** |
| Serotype |  |  |  |  |  |  |  | 0.92 |
| - DENV-1 | 9/62 | NA (NA, NA) |  | 10/53 | NA (NA, NA) | 1.4 (0.6, 3.5) | 0.46 |  |
| - DENV-2 | 4/18 | NA (NA, NA) |  | 7/15 | NA (5.0, NA) | 2.6 (0.5, 12.2) | 0.24 |  |
| - DENV-3 | 8/13 | 5.0 (4.0, NA) |  | 8/15 | 5.0 (4.0, NA) | 1.6 (0.5, 5.6) | 0.44 |  |
| - DENV-4 | 50/52 | 3.0 (2.0, 4.0) |  | 62/64 | 3.0 (2.0, 4.0) | 1.3 (0.8, 1.9) | 0.26 |  |
| Serology |  |  |  |  |  |  |  | 0.21 |
| - Primary | 12/33 | NA (4, NA) |  | 18/43 | NA (4.0, NA) | 0.5 (0.2, 1.2) | 0.11 |  |
| - Secondary | 58/109 | 5.0 (3.0, NA) |  | 64/94 | 4.0 (3.0, NA) | 1.5 (1.0, 2.2) | 0.03 |  |

These analyses were based on Cox regression with adjustment for day of illness at enrolment, baseline log10 viremia, serotype and serology. Heterogeneity was assessed with a likelihood-ratio (chi-squared) test for an interaction term between treatment group and sub-grouping variable in the statistical model.

Median (IQR) time to undetectable viremia was based on Kaplan-Meier estimation. “NA” indicates that values could not be estimated because not enough subjects achieved undetectable viremia. “Events” refers to cases who achieved undetectable viremia.
